# 816. Fatal Coccidioidomycosis Cases in an Endemic Area

**DOI:** 10.1093/ofid/ofad500.861

**Published:** 2023-11-27

**Authors:** Carlos M D’Assumpcao, Lovedip Kooner, Amritpal Dhillon, Michael Valdez, Rasha Kuran, Royce H Johnson, Arash Heidari

**Affiliations:** Kern Medical, Bakersfield, California; Valley Fever Institute at Kern Medical, Bakersfield, California; Kern Medical, Bakersfield, California; Kern Medical, Bakersfield, California; Kern Medical Center, Bakersfield, California; Kern Medical Center, Bakersfield, California; Dignity Health, Bakersfield, CA

## Abstract

**Background:**

While a majority of *Coccidioides* infections are asymptomatic, it is estimated that 1% develop severe disease and can be fatal. Prior retrospective reviews that attempt to identify risk factors for fatal disease have been limited to public health data hampered by reporting challenges and accuracies. Morbidity review of fatal cases identified by clinical criteria rather than ICD-9/ICD-10 reporting may improve accuracy of conclusions.

**Methods:**

At an academic center in an endemic area, medical records from three sequential electronic medical record systems were reviewed from approximately January 2000 to January 2023. Patients with coccidioidomycosis were identified by microbiological, pathological, serological, or skin testing criteria. Deaths were determined by in hospital records, insurance reporting, review of death certificate if available, or public record. Demographics, clinical course, outcomes and causes of death are compared. Incomplete medical records to make comparison were excluded. Retrospective root cause analysis was performed.

**Results:**

To date, at least 50 patients met clinical criteria for having coccidioidomycosis at one point in their life and died. ICD coding was wrong in about half of the cases. Data integrity has been a challenge when extracting data across three electronic medical record systems. New risk factors found by root cause analysis not previously identified.

**Conclusion:**

Patients were found to have died either with coccidioidomycosis or from coccidioidomycosis. ICD coding was inconsistent. Precise and accurate description of coccidioidomycosis staging is important when trying to determine risk factors for fatal disease.

**Disclosures:**

**All Authors**: No reported disclosures

